# Experimental concepts for toxicity prevention and tissue restoration after central nervous system irradiation

**DOI:** 10.1186/1748-717X-2-23

**Published:** 2007-06-30

**Authors:** Carsten Nieder, Nicolaus Andratschke, Sabrina T Astner

**Affiliations:** 1 Radiation Oncology Unit, Nordlandssykehuset HF, 8092 Bodø, Norway; 2 Department of Radiation Oncology, Klinikum rechts der Isar der Technischen Universität München, Ismaninger Str. 22, 81675 Munich, Germany

## Abstract

Several experimental strategies of radiation-induced central nervous system toxicity prevention have recently resulted in encouraging data. The present review summarizes the background for this research and the treatment results. It extends to the perspectives of tissue regeneration strategies, based for example on stem and progenitor cells. Preliminary data suggest a scenario with individually tailored strategies where patients with certain types of comorbidity, resulting in impaired regeneration reserve capacity, might be considered for toxicity prevention, while others might be "salvaged" by delayed interventions that circumvent the problem of normal tissue specificity. Given the complexity of radiation-induced changes, single target interventions might not suffice. Future interventions might vary with patient age, elapsed time from radiotherapy and toxicity type. Potential components include several drugs that interact with neurodegeneration, cell transplantation (into the CNS itself, the blood stream, or both) and creation of reparative signals and a permissive microenvironment, e.g., for cell homing. Without manipulation of the stem cell niche either by cell transfection or addition of appropriate chemokines and growth factors and by providing normal perfusion of the affected region, durable success of such cell-based approaches is hard to imagine.

## Background

The risk of permanent central nervous system (CNS) toxicity, which typically becomes detectable after an asymptomatic latency period, continues to influence clinical treatment decisions. Interindividual differences in sensitivity result in a certain variability of the threshold dose and preclude administration of a guaranteed safe dose, even in the current era of high-precision image-guided radiotherapy. The easiest and most effective way of avoiding CNS side effects is to minimize the dose of radiation. This does, however, not solve the problem of normal tissue present within the target volume, for example due to diffuse microscopic spread, which escapes current imaging technology. For certain groups of patients, further progress can only be expected from efforts directed at widening the therapeutic window between tumor and normal tissue through specific modulation of their responses to radiotherapy (e.g., toxicity prevention) or from delayed intervention such as tissue regeneration strategies. Both prevention and treatment of side effects have their specific advantages and disadvantages. Importantly, they are not standard clinical options at this time. To exploit potential targets for intervention, we will discuss the pathogenesis of radiation-induced CNS toxicity and review preclinical data on prevention and tissue regeneration. We focus on two types of damage, i.e. neurocognitive decline and radiation necrosis. The latter is relevant to treatment of the brain and the spinal cord.

## Pathogenesis

Initial evaluations of radiation-induced CNS toxicity date back at least 70 years ago. These historical data have been summarized in previous reviews, for example by van der Kogel [1] and Schultheiss et al. [2]. In brief, previous experimental studies indicated that signs of diffuse demyelination develop in animals 2 weeks after CNS radiotherapy. After approximately 2 months, remyelination processes were observed. These early changes correspond to clinical symptoms such as Lhermitte's sign and somnolence in humans. After a variable latency period, and dependent on total dose, white matter necrosis might develop. The grey matter is less sensitive. Latency time decreases with increasing radiation dose. The most important determinants of CNS tolerance are the volume of normal tissue exposed, dose per fraction and total dose. Overall treatment time is less important. With multiple fractions per day, incomplete repair needs to be taken into account, especially when the interfraction interval is less than 6 h. When high focal doses are combined with lower doses to a large surrounding volume, tolerance decreases compared to the same focal treatment alone.

Significant long-term recovery has been observed after spinal cord radiotherapy. Although not experimentally tested in the same fashion, it can be assumed that the brain recovers too. Especially with larger intervals of at least 1–2 years and when the first treatment course was not too close to tolerance, re-irradiation is now considered as a realistic option. Experimental data from fractionated radiotherapy of rhesus monkeys suggest that up to 75% of initial damage recover within 2–3 years [3]. Increasing clinical evidence supports the feasibility of re-irradiation in selected patients [4].

The last years have witnessed a significant improvement of techniques in cellular and molecular biology, resulting for example in description of more and more radiobiologically relevant signalling pathways [5]. Advanced methods for identification of stem and progenitor cells were developed. Meanwhile, this progress has led to a better understanding of tissue responses to ionizing radiation. Obviously, radiation-induced reactions of the CNS include death of both immature and mature parenchymal and vascular cell populations, executed via different mechanisms at different time points. Apoptosis induced by sphingomyelinase-mediated release of ceramide has been described as early reaction in endothelial cells within the irradiated CNS [6,7] as well as in oligodendrocytes [8]. Current models of radiation-induced changes include a cascade of complex and dynamic interactions between mature parenchymal cells (oligodendrocytes, astrocytes, microglia, neurons), stem and progenitor cells and the vascular system, also resulting in important alterations of the local microenvironment [9]. The latent time preceding the clinical manifestation of damage is viewed as an active phase where chemokines, cytokines and growth factors play important roles in intra- and intercellular communication.

CNS radiotherapy induces the production of inflammatory cytokines and mediators such as tumor-necrosis-factor-α (TNF-α), interleukin-1 (IL-1), and prostaglandin E2 by microglia and astrocytes [10-12]. Some of these facilitate transendothelial migration of immune cells. IL-1 release leads, via autocrine mechanisms, to further activation and proliferation of these glia cells. As shown *in vivo*, this cascade results in astrogliosis [13]. Furthermore, inflammatory microenvironmental changes can impair the compensation of the radiation-induced cell loss. TNF-α is also known to damage endothelial cells, leading to increased vascular permeability. TNF-α and IL-1 induce the expression of intercellular adhesion molecule-1 (ICAM-1) on oligodendrocytes and microvascular endothelial cells [14,15]. Increased levels of ICAM-1 mRNA were detectable after midbrain irradiation with 2 Gy [16]. Results of localized single-fraction treatment with 20 Gy confirm the presence of an early inflammatory response, increased numbers of leukocytes, increased vascular permeability, altered integrity of endothelial tight junctions and increased cell adhesion [17,18]. Injection of an anti-ICAM-1 monoclonal antibody significantly reduced leukocyte adhesion and permeability in this model. The role and time course of inflammatory mediators varies with fraction size. Certainly, the cellular and molecular events during the latent phase require further research. The role of TNF, for example, might be more complex than initially thought. In some models, this cytokine mediates antioxidant defense mechanisms and is able to induce antiapoptotic proteins such as Bcl-2. Furthermore, TNF-receptor-p75 knockout mice were more sensitive against radiation-induced brain damage than control mice and TNF-receptor-p55 knockouts [19].

### Special aspects of neurocognitive deficits

Phenomena such as intellectual decline and memory loss in the absence of gross perfusion disturbance suggest that neuronal cells react to radiotherapy. Experimental studies have demonstrated that neurons and precursor cells might undergo apoptosis after radiotherapy [20]. Fractionated brain irradiation inhibited the formation of new neurons in the dentate gyrus of the hippocampus in rats [21]. Animals with blocked neurogenesis performed poorer in short-term memory tests which are related to hippocampal function. The deficit in neurogenesis is based on both reduced proliferative capacity of progenitor cells and alterations in the microenvironment that regulates progenitor cell fate (disruption of the microvascular angiogenesis, activation of microglia) [22]. After higher doses of whole-brain radiotherapy (WBRT, 8 fractions of 5 Gy) in rats, cognitive impairment arose after a significant loss of brain capillaries [23], suggesting once more a multifactorial pathogenesis. The latter might also include changes in hippocampal glutamate receptor composition, as recently suggest by Shi et al. [24].

### Special aspects of radiation necrosis of the brain and spinal cord

Initial events are similar to those described in the pathogenesis section, including inflammatory changes and increased vessel permeability. Studies of boron-neutron-capture therapy (BNCT) support the view that vascular damage is one of the crucial components leading to radiation necrosis after higher doses. By choosing boron-compounds which are unable to cross the blood-brain barrier, a largely selective irradiation of the vessel walls can be accomplished with BNCT. Compared to conventional non-selective radiotherapy methods, spinal cord lesions with similar histological appearance were induced. Latency time also was comparable between damage induced by BNCT and conventional radiotherapy [25,26]. Additional evidence is provided by histological examinations of rat brains after radiotherapy with 22.5 or 25 Gy, showing reduced numbers of blood vessels and endothelial cells before manifestation of necrosis [27]. A study in rats (partial brain irradiation with 40 or 60 Gy or WBRT with 25 Gy) showed a 15% reduction in endothelial cell number between 24 h and 4 weeks after radiotherapy. A further reduction was seen with even longer intervals [28]. Theses changes are accompanied by hyperpermeability, resulting in perivascular edema and consecutive ischemic damage [29].

Kamiryo et al. showed how the latency to development of vascular damage after stereotactic radiosurgery (SRS) to the parietal cortex of rat brain decreases from 12 months to 3 weeks with an increase in radiation dose from 50 to 75 or 120 Gy [30]. The amount of vessel dilation, increased permeability, thickening of the vessel wall, vessel occlusion and necrosis also increased with dose. Spinal cord data suggest an increase in the release of vascular endothelial growth factor (VEGF) as a result of impaired perfusion and hypoxia signalling [31]. Obviously, the clinically observed latent phase is characterised by persistent and increasing oxidative stress and active responses to this factor.

## Clinical confirmatory data

Sustaining toxicity that may impair the patients' lifestyle significantly can be observed several years after radiotherapy in form of radionecrosis and cognitive dysfunction associated with leukoencephalopathy. Necrosis develops mostly after 1–3 years [32]. The typical finding is coagulation necrosis in the white matter with largely normal appearance of the cortex. Fibrinoid necrosis and hyalinous wall thickening of blood vessels are commonly observed. Therapeutic intervention with corticosteroids or anticoagulants is sometimes successful. Often, surgical resection is the only way to effectively improve the symptoms.

Diffuse white matter changes are frequently observed in imaging studies. Fluid-attentuated inversion recovery (FLAIR) and diffusion-weighted MRI might improve visualization of white matter abnormalities, which are not necessarily associated with clinical symptoms but often present after fractionated doses of ≥ 30 Gy. Neuropsychological sequelae typically manifest within 4 years from radiotherapy. Psychometric findings suggest greater vulnerability of white matter and subcortical structures resulting in reduced processing speed, heightened distractability and memory impairment. Within the temporal lobe, the hippocampal formation plays a central role in short-term memory and learning. These functions are related to the activity of neural stem cells. The hippocampal granule cell layer undergoes continuous renewal and restructuring. Radiotherapy can affect this sensitive cell layer leading to impaired function without overt pathological changes.

There is increasing evidence that partial brain radiotherapy alone rarely causes significant neurocognitive decline [33,34]. One of the largest comparative studies in low-grade glioma showed poorer cognitive function in irradiated patients [35]. However, cognitive disability was associated to fraction doses exceeding 2 Gy. In addition, antiepileptic drug use was strongly associated with disability in attentional and executive function. The risk of toxicity might also increase with age, probably as a result of impaired tissue reserve capacity and perfusion. Increased sensitivity of children might be related to conditions in the immature CNS, e.g., increased proliferation. Neurocognitive dysfunction was reported to stabilize spontaneously [36] or to progress over time [37]. In extreme cases, subcortical dementia might result which often is associated with gait disturbance and incontinence. Due to the lack of effective treatment, most patients with this severe complication die after several months or a few years. Histopathologic findings include diffuse spongiosis and demyelination as well as dissiminated miliar necrosis.

## Prevention strategies

At present, pharmacologic or biologic prevention approaches are still considered experimental, despite of some non-randomized trials, e.g., of SRS for arteriovenous malformations where patients treated with gamma linolenic [omega-6-] acid had less permanent complications than those who did not receive this medication [38]. However, several rational experimental interventions based on the pathogenetic models reviewed earlier have been studied or are currently under investigation. The clinical effectiveness of these putative prevention strategies has yet to be established.

**Figure 1:**
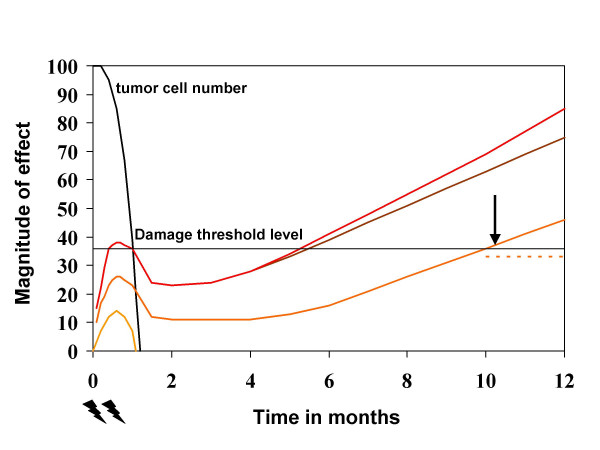
Schematic concept of the time course of radiation-induced reactions in cancer patients treated with ionizing radiation via portals exposing some part of the central nervous system (CNS). The tumor is expected to become eradicated within a few weeks. The severity and latency of CNS reactions are dose-dependent. Three different levels are shown. Acute CNS reactions often remain below the level of clinical detection and resolve early. A second wave of so-called late reactions might develop after several months or years and after higher radiation doses. The upper curve with or without additional comorbidity shows how certain factors might influence damage progression or make intervention more difficult. The dotted line below the threshold level represents succesful therapeutic intervention, which was started at the time indicated by the arrow.

On the one hand, the multifactorial pathogenesis offers many different targets for intervention [39], on the other hand targeting just one of these complex cascades might not be sufficient to effectively inhibit tissue degeneration. Figure 1 illustrates that early intervention has to deal with functional rather than structural and clinically manifest damage. While early-stage damage might be easier to treat, any intervention faces the challenge of selectivity or the risk of tumor protection. Among the earliest events that might be targeted are direct and indirect radiation effects leading to DNA damage. Indirect effects, mediated via reactive oxygen species, can be counteracted by radical scavengers such as amifostine. Several independent experiments with different endpoints, illustrated in Table 1, provided preliminary evidence that modulation of the radiation response of the CNS in vivo by systemic administration of amifostine appears possible. However, additional studies are warranted to investigate the protective effect with differing regimens of administration, more clinically relevant fractionation regimens, and longer follow-up. Various other compounds are also able to interact with free radicals, for example glutathione. With any of these agents, complete dose-effect curves have yet to be generated to firmly establish their role in prevention.

**Table 1:** Overview of experimental studies of central nervous system (CNS) radioprotection

Reference	Animals	CNS region	RT schedule	AF schedule	Follow-up	Results
Guelman et al. [88]	Neonatal Wistar rats	Cephalic end	1 × 5 Gy	Subcutaneously 100 mg/kg	30 days (90 days for 1 endpoint)	Sign. protection
Alaoui et al. [89]	Young Sprague-Dawley rats	Whole body (brain)	1 × 2.5 Gy	Intraperitoneal 75 mg/kg	6 hours	No sign. protection
Lamproglou et al. [90]	Young Wistar rats	Whole brain	10 × 3 Gy	Intraperitoneal 37.5, 75 and 150 mg/kg	7.5 months	37.5 mg/kg not effective; 150 mg/kg caused 34% mortality; 75 mg/kg reduced memory dysfunction
Plotnikova et al. [91, 92]	Adult Wistar rats	Whole brain	1 × 25 Gy (earlier study with 40 or 60 Gy)	Intraperitoneal 300 mg/kg	18 months	Protection against vascular damage, necrosis and death after 25 Gy only
Spence et al. [93]	Adult F-344 rats	Spinal cord	1 × 20–38 Gy	Intrathecal 0.33 mg	36 weeks	Protection with DMF 1.3
Nieder et al. [94]	Adult F344 rats	Spinal cord	2 fractions, high dose	Intrathecal 0.3 mg	12 months	No sign. protection
Nieder et al. [94]	Adult F344 rats	Spinal cord	2 fractions, high dose	Subcutaneous 200 mg/kg	12 months	Protection at 36 Gy-level
Nieder et al. [43]	Adult F344 rats	Spinal cord	Single fraction, high dose	Intrathecal 0.3 mg plus s.c. IGF-1	12 months	Protection with DMF 1.07
Andratschke et al. [44]	Adult F344 rats	Spinal cord	2 fractions, high dose	Intrathecal PDGF as sole treatment	12 months	Protection with DMF 1.05

RT: radiotherapy; AF: amifostine; IGF-1: insulin-like growth factor-1; PDGF: platelet-derived growth factor; DMF: dose modification factor

DNA damage repair can be enhanced by several compounds, including the growth factor insulin-like growth factor-1 (IGF-1) [40]. As demonstrated by our group, s.c. IGF-1 treatment for few days concomitant to irradiation significantly increases the latent time to development of spinal cord necrosis [41]. When combined with intrathecal basic fibroblast growth factor (FGF-2) or amifostine, a better efficacy was observed [42,43]. Dose-effect curves were generated only for the combination of s.c. IGF-1 with intrathecal amifostine. They suggest an increase in the long-term radiation tolerance by approximately 7% for single fraction irradiation. Growth factors, however, might also influence several other mechanisms. They were shown to prevent radiation-induced apoptosis, influence proliferation of stem cells, neurogenesis and angiogenesis. Pena et al. have shown that i.v. injections of FGF-2 5 min. before, immediately after and 1 h after total body irradiation in mice (1–20 Gy or 50 Gy) significantly reduced the number of apoptotic vascular and glial cells in the CNS [6]. Spinal cord experiments suggest that other growth factors, such as platelet-derived growth factor (PDGF) can increase the long-term radiation tolerance by approximately 5% (two fractions of 16–20 Gy 24 h apart, PDGF given intrathecally for 4 days starting 24 h before the first fraction of radiation) [44]. It has recently been suggested that i.p. injections of carbamylated erythropoietin, which does not stimulate the bone marrow, reduce the extent of brain necrosis in rats exposed to a single dose of 100 Gy (administration for 10 days starting immediately before radiosurgery [45]). Thus, several experiments demonstrated that delayed toxicity can be prevented by early intervention at the time of radiation treatment. This offers new strategies of toxicity prevention. It was also suggested that growth factors have bell-shaped dose-effect curves, i.e. high doses do not exert the best effects. Moreover, high doses of PDGF or VEGF might even cause acceleration of damage expression, most likely via cell-cycle-activating signals [46]. Usually, many cell types undergo p53-induced G1-arrest after radiotherapy to allow for repair of treatment-induced lesions. By overriding this mechanism with high doses of growth factors, such cells might be forced to die, resulting in early tissue breakdown and manifestation of damage. With delayed treatment after 12 weeks or more, acceleration was no longer observed, suggesting that the damage cascade might already have reached a stage where additional manipulation can not influence the outcome anymore.

Whether growth factors influence pathways leading to neurocognitive deficits is less well studied. Fukuda et al. suggested that erythropoietin (EPO) did not influence single-dose irradiation-induced cell death in the dentate gyrus of immature rodents [47]. However, neurocognitive testing was not performed. Hossain et al. confirmed that EPO did not modify the apoptotic response in this region in adult mice treated with single-dose WBRT [48]. EPO also did not reverse the inhibition of neurogenesis. However, reduced expression of inflammatory genes such as COX-2 and ICAM-1 in the hippocampus was observed.

Several other examples of inhibition of inflammatory reactions are available. The prophylactic use of dexamethasone 24 and 1 h before radiation exposure reduced the expression of TNF-α, IL-1 and ICAM-1 [16]. *In vitro*, corticosteroids influence the function of microglial cells and inhibit their proliferation [49]. Kondziolka et al. irradiated rats with implanted cerebral glioma by SRS, either with or without i.v. administration of U-74389G, a 21-aminosteroid which is largely selective for endothelium [50]. The compound reduced the development of peritumoral edema and of radiation-induced vascular changes in the parts of the brain which were within the region of the steep dose gradient outside of the target volume. Injection of an anti-ICAM-1 monoclonal antibody significantly reduced leukocyte adhesion and vessel permeability in a different rat model [18]. Monje et al. observed a decrease in activated microglia and proliferating peripheral monocytes and an increase in newborn hippocampal neurons in adult rats treated with a single dose of 10 Gy and daily indomethacin for 2 months beginning 2 days before brain irradiation [51]. Compared to animals that did not receive radiation, neurogenesis was still limited to 20–25%. No functional endpoints were reported. Recently, Zhao et al. described a rat model of fractionated WBRT with or without pioglitazone, an anti-inflammatory peroxisomal proliferator-activated receptor gamma agonist [52]. The WBRT-induced cognitive impairment was best prevented by drug administration before, during, and after WBRT. Thus, preliminary data suggest protection from neurocognitive damage or necrosis with anti-inflammatory drugs, but dose-modification factors have not been generated yet.

## Delayed intervention/treatment of side effects/tissue restoration

As suggested in Figure 1, delayed intervention during the latency time circumvents the problem of tumor protection. However, trying to reverse or ameliorate side effects will only be possible before a certain threshold level of damage is exceeded. Higher radiation doses might require either earlier or more efficacious interventions. In addition, comorbidity associated with perfusion disturbance might modify damage progression. A few case reports described successful treatment of late CNS toxicity by hyperbaric oxygen treatment (HBO). For example, one out of 7 patients with cognitive impairment at least 1.5 years after radiotherapy improved after 30 sessions of HBO [53]. Patients with leukencephalopathy and moderate hydrocephalus (diagnosed by intracranial pressure monitoring) might profit from ventriculoperitoneal shunt insertion [54]. Quality of life can be improved by supportive measures (cognitive training, rehabilitation, special education etc.) and possibly by drugs prescribed for other neurodegenerative diseases or depression [55]. Some of these compounds such as fluoxetine increase neurogenesis [56]. For radionecrosis of the brain, therapeutic intervention with corticosteroids or anticoagulants is sometimes successful. They should be administered early before the stage of cystic liquefaction. Often, surgical resection is the only way to effectively improve the symptoms. Very recent, preliminary data suggest that VEGF pathway inhibition with bevacizumab might be able to reduce both the MRI abnormalities associated with necrosis and the dexamethasone requirement [57]. These findings lend support to the preclinical spinal cord radionecrosis data [31].

Ramipril, an inhibitor of angiotensin-converting enzyme, was studied in a rat model of optic neuropathy 6 months after irradiation with both functional and histological endpoints [58]. Continuous daily drug treatment started already 2 weeks after irradiation. Encouraging results for both endpoints were reported. However, only a single radiation dose level was examined. Hornsey et al. evaluated vasoactive drugs administered from 17 weeks onwards after single-dose irradiation of rat spinal cord [59]. Dipyridamol increased the median latent time from 167 to 195 days at the level of the ED_100 _and from 193 to 240 days at the ED_80_. Moreover, the better effectiveness at lower radiation doses led to an increase in ED_50 _by 2–3 Gy (approximately 10%).

Transplantation of stem cells or stimulation of the endogenous stem cell compartment, e.g., by growth factor application might also offer exciting prospects. In principle, mature functional cells can be generated by proliferation and differentiation from stem, progenitor, and precursor cells or by recovery and repair of damage in already existing cells which then continue to survive. Important differences exist between embryonic, umbilical cord blood, and various types of adult stem cells. All of these, however, are capable of self-renewal, a process by which stem cells divide to generate one (asymmetric division) or two (symmetric division) daughter stem cells, are proliferative, and are multipotent for the different cell lineages. Besides of killing stem cells, ionizing radiation could also exert adverse effects if it would directly or indirectly change the programming and behaviour of these cells, e.g., by triggering generation of glial cells only or by maintaining their own stem cell pool without generation of differentiated progeny. Stem cell maintenance, prevention of premature senescence and apoptosis, and differentiation in the mammalian CNS are complex and well regulated, e.g., by Sonic Hedgehog, Polycomb family members, cell cycle regulators, and environmental factors in the stem cell niche [60,61].

**Figure 2:**
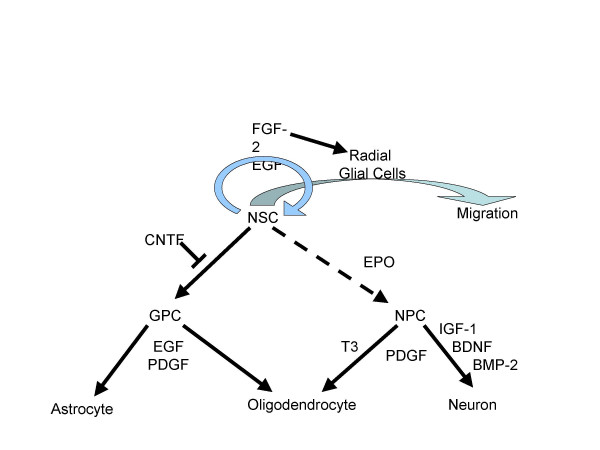
Growth factors influence several steps of neurogenesis. NSC: neural stem cell, NPC: neural progenitor cell, GPC: glial progenitor cell, FGF-2: basic fibroblast growth factor, EGF: epidermal growth factor, CNTF: ciliary neurotrophic factor, EPO: erythropoietin, PDGF: platelet-derived growth factor, IGF-1: insulin-like growth factor-1, BMP-2: bone morphogenetic protein-2, BDNF: brain-derived neurotrophic factor, T3: thyroid hormone

Both hematopoietic and neural stem cells might be beneficial for CNS regeneration. Neural stem cells can be divided into two different subsets, i.e. CNS stem cells and neural crest stem cells. The latter give rise to neurons and glia of the peripheral nervous system and other connective cell types. The subventricular zones (SVZ) adjacent to the lateral ventricles contain a mosaic of immature multipotential, bipotential, and unipotential neural CNS stem cells as well as progenitors at different stages of lineage restriction (Figure 2). Other regions in the adult CNS, incl. hippocampus, optic nerve and spinal cord, contain at least certain types of precursors (reviewed by Emsley et al. [62]). Several growth factors instruct lineage differentiation. In addition, there are switches, such as Notch activation, that determine neurogenesis, which normally occurs first, and initiate gliogenesis. Some of these CNS precursor cells are highly sensitive to ionizing radiation and undergo apoptosis, as already discussed. Interestingly, neural stem cells are less prone to apoptosis as progenitors, e.g. late oligodendrocyte progenitors (reviewed by Romanko et al. [63]). Tada et al. showed that 24 h after irradiation of rat brains significant reductions occur in total cell number, and in the number of proliferating cells and immature neurons in the SVZ [64]. With higher radiation doses no relevant repopulation of the SVZ was observed for at least 6 months. Obviously, surviving stem cells do not receive the proper signals to initiate tissue recovery after irradiation or maybe surrounding supportive elements are lost (see inflammatory and vascular changes reviewed earlier). Another limiting factor for endogenous stem cells is the fact that they undergo cell-intrinsic changes in developmental or neuronal subtype potential over time [65], possibly reducing their capacity to form neurons and biasing the types of neurons they can make. It can not be excluded that radiation-induced gliosis might prevent generation of the required cell types [61]. Furthermore, activation of both neural and endothelial/vascular cell lineages might be required to achieve durable success. Neural stem cells grown with endothelial cells *in vitro *underwent symmetric, proliferative divisions, in contrast to the asymmetric pattern seen in control conditions [66]. Endothelial cells secrete factors such as FGF-2 that influence self-renewal and neurogenic potential. While the stem cells generated neurons, astrocytes, and oligodendrocytes upon endothelial cell removal, no endothelial progeny was generated.

Immature cells are able to migrate tangentially and radially within the CNS for a limited distance, possibly leading to regeneration of small lesions from the surrounding healthy tissue [67]. Astrocytes and endothelial cells up-regulate chemokines such as stromal cell-derived factor (SDF)-1α after injury. As shown by Imitola et al., neural stem cells by virtue of their expression of chemokine receptors migrate to sources of SDF-1α and home to the injury-induced stem cell niches [68]. Migration also depends on adhesion and extracellular matrix molecules. Without manipulation, there appears to be limited directed cell migration and replacement from endogenous cell pools, e.g., in the SVZ. Growth factors might represent potential tools for manipulation. Different experimental CNS damage models suggest that IGF-1 causes an increase in oligodendrocyte numbers in previously damaged areas of the rat spinal cord [69]. IGF-1 reduces the permeability of the blood-brain barrier and has been found to influence the restoration of neurogenesis in the adult and aging hippocampus [70]. Granulocyte colony-stimulating factor (G-CSF) also induced proliferation and differentiation of neural precursors and endothelial cell proliferation in adult rat brain *in vivo*, most likely via VEGF interaction [71]. Brain-derived neurotrophic factor (BDNF) also leads to recruitment of endothelial cells and increase of capillary density [72]. However, even in theory finding the right dose, timing and maybe combination and sequence of different growth factors in an individual patient appears very challenging, not to mention that growth factor doses in some experimental conditions are too high for human application. Limited time intrathecal administration of VEGF or PDGF for two weeks starting 8–16 weeks after rat spinal cord irradiation was not effective in preventing necrosis (own unpublished data), underlining that relatively simple interventions aiming at the surviving endogenous cell population might not be the preferable approach in a complexly altered CNS environment.

As an alternative, exogenous neural stem cells might induce tissue regeneration. Such cells can even be engineered to manipulate their own microenvironment, as shown for example by Zhu et al. who transfected fetal neural stem cells with VEGF gene [73]. After transplantation, the stem cells migrated and expressed VEGF during the early time after transplantation. Later, some of them differentiated to neurons. If precursor cells rather than stem cells are transplanted into neurogenic regions, they can differentiate into neurons in a region-specific manner [74]. When transplanted outside the neurogenic regions, they might generate only glia [62]. Thus, neurogenesis is dependent on a permissive microenvironment. This again leads to the question of how neurogenic permissiveness can be induced or modified because donor cells, whatever their source, must interact with an extremely complex CNS environment in order to integrate appropriately. The same holds true for the other main endpoint, i.e. radiation necrosis. O-2A progenitor cells transplanted into irradiated rat spinal cord were shown to divide, migrate and contribute to remyelination [75]. Rezvani et al. used neural stem cell transplantation to protect rats against spinal cord necrosis [76]. Their results were encouraging, however, follow-up was shorter than 12 months. Furthermore, they conducted the study in younger rats whose immature spinal cord might react differently.

What results can be expected from transplanted non-neural cells? A detailed description of this issue is beyond the scope of this paragraph, as recent reviews provide a lot of background information, e.g. [77]. Umbilical cord blood-derived cells have been identified in the CNS and endothelium [78] and were beneficial in a mouse model of amyotrophic lateral sclerosis [79]. It has been suggested, however, that hematopoietic stem cells maintain lineage fidelity in the brain and do not adopt neural cell fates [80] or transdifferentiate [81]. We are not aware of studies having addressed this question specifically in irradiated CNS. A French group transplanted human mesenchymal stem cells into mice subjected to sublethal total body irradiation (TBI) with or without superimposed local fields [82,83]. Without irradiation, these stem cells did not engraft in the brain within 15 days (maximum observation time). After TBI increased engraftment was detected. In a model of mouse skin irradiation, beneficial effects of cultured bone marrow mesenchymal stem cells on lesion healing were suggested too [84]. With regard to experimental conditions, it has to be emphasized that observations in precursor research in general might be site- and condition-specific and thus hard to generalize. Some of the observations still create considerable controversy (fact or artifact, as reviewed by Krabbe et al. [85]). It should also be mentioned that stimulation of precursor cell proliferation does not necessarily lead to sufficient numbers of those differentiated cells that keep the organ functional. This is emphasized by observations of lack of differentiation of O-2A cells into oligodendrocytes [86] and differentiation of endothelial progenitors into smooth muscle cells, potentially increasing the thickness of the blood vessel wall [87] after treatment with PDGF-BB. It is also clear that true neuronal integration depends on many complex variables and progressive events.

## Conclusion

Although a large body of research on radiation-induced CNS toxicity is still necessary, one can envision a scenario with individually tailored strategies where patients with comorbidity resulting in impaired regeneration reserve capacity might be considered for toxicity prevention, while others might be "salvaged" by delayed interventions that circumvent the problem of normal tissue specificity. Given the complexity of radiation-induced changes, single target interventions might not suffice. Intervention might vary by patient age, elapsed time from radiotherapy and toxicity type. Potential components include drugs that target neurodegeneration or perfusion/hypoxia, cell transplantation (into the CNS itself, the blood stream, or both) and creation of reparative signals and a permissive microenvironment, e.g., for cell homing. Without manipulation of the stem cell niche either by cell transfection or addition of appropriate chemokines and growth factors and by providing normal perfusion of the affected region, durable success of cell-based strategies is hard to imagine.

## Competing interests

The author(s) declare that they have no competing interests.

## Authors' contributions

CN and NA participated in the conception of the work. NA and STA performed data acquisition and interpretation. CN drafted the manuscript. All authors read and approved the final manuscript.
